# Reply to the Letter to the Editors by Beirão

**DOI:** 10.1590/0102-311XEN073224

**Published:** 2024-06-21

**Authors:** Akira Homma, Marcos da Silva Freire, Cristina Possas

**Affiliations:** 1 Instituto de Tecnologia em Imunobiológicos, Fundação Oswaldo Cruz, Rio de Janeiro, Brasil.

In his interesting letter to the Editors on our article on vaccines for neglected and emerging diseases ^1^, Prof. Beirão ^2^ makes incisive comments and a very positive evaluation of our publication. He also gives important considerations on the main challenges remaining for Brazil to be able to come close to overcoming the “valley of death” in vaccine development by 2030. In summary, he concludes that the COVID-19 pandemic has had “*the positive impact of pushing Brazilian R&D towards the goals*”, but alerts that “*now is the time to keep up the momentum to be able to come close of reaching the goals set in the article*” ^2^ (p. 2).

The struggle of the pandemic has had the positive impact of pushing Brazilian R&D towards the goals of our article, but now he ponders that it is time to keep up the momentum to be able to come close of reaching the goals set in the article.

We agree with most of his observations and alerts, but have some critical comments as indicated below. As Prof. Beirão has stressed, this article was written in 2019, before the pandemic. Our recent publications ^3^ ^,^ ^4^ clarify many of the issues he discusses in his letter.

The severe dengue epidemic and partial success with vaccines (in spite of very low vaccine availability in the country so far) has certainly stimulated vaccine research against the disease and other neglected and emerging diseases that affect the Brazilian population. Nevertheless, the main obstacle to boost immunization against these diseases has been the lack of adequate governance and sustained financial support. It is true, as the letter mentions, that the progress within the country is necessary, but national research institutions have so far received very limited funding to achieve the conditions necessary to accelerate vaccine innovation and development. As mentioned in the letter, research on these pathogens relies on developing countries that suffer from them. However, these diseases do not attract pharmaceutical enterprises attention due to their limited market scope, which has important constraints to innovation and technological transfer from these enterprises to developing countries. In spite of governmental efforts, there are still few public-private partnerships for product development.

We do not agree that the percentage of gross domestic product (GDP) invested in research, development and innovation (RD&I) in Brazil is reasonable, considering the country’s historical debt with innovation and technological development since the 1988 Constitution and its place as the 8th economy of the world. Lack of adequate and sustained funding for vaccine innovation in Brazil and of a consistent science and technology policy has been an historical issue and a major obstacle for building self-sufficiency in this area. The external dependence is extremely high: Brazil still imports 90% of the inputs used to make vaccines and medicines ^5^.

We also do not agree that insufficient awareness on poverty is an issue among Brazilian scientists. Most researchers in Brazil are socially-responsible and aware of the need to deliver outputs that are directly beneficial to the poorest populations. Researchers and research institutes should not be blamed for requiring more resources: they demand increased participation of RD&I in GDP and prompt non-bureaucratic access to these resources, since delivering these outputs to society is impossible without adequate governance and sustained funding. In an era of Vaccinology 4.0, with new vaccines emerging as complex multipatented products ^6^ and requiring in many cases innovative mRNA platforms, this national scenario of virtual lack of funding becomes a critical issue. Achieving an adequate vaccine policy and an exponential increase in sustained funding should thus be our main strategic goal.

We agree that there are other issues besides funding: a National Vaccine Authority promoting knowledge governance and synergy among all vaccine research institutes is urgently needed, with the mission to coordinating policy, capacity building, infra-structure, and funding mechanisms in the direction of the UN 2030 Agenda for Sustainable Development.
